# Evasion of Innate and Intrinsic Antiviral Pathways by the Zika Virus

**DOI:** 10.3390/v11100970

**Published:** 2019-10-22

**Authors:** Taryn M. Serman, Michaela U. Gack

**Affiliations:** Department of Microbiology, The University of Chicago, Chicago, IL 60637, USA; taryn@uchicago.edu

**Keywords:** flavivirus, Zika virus, innate immunity, antiviral, interferon, stress response, nonsense-mediated mRNA Decay, autophagy

## Abstract

The Zika virus (ZIKV) is a recently emerged mosquito-borne flavivirus that, while typically asymptomatic, can cause neurological symptoms in adults and birth defects in babies born to infected mothers. The interactions of ZIKV with many different pathways in the human host ultimately determine successful virus replication and ZIKV-induced pathogenesis; however, the molecular mechanisms of such host-ZIKV interactions have just begun to be elucidated. Here, we summarize the recent advances that defined the mechanisms by which ZIKV antagonizes antiviral innate immune signaling pathways, with a particular focus on evasion of the type I interferon response in the human host. Furthermore, we describe emerging evidence that indicated the contribution of several cell-intrinsic mechanisms to an effective restriction of ZIKV infection, such as nonsense-mediated mRNA decay, stress granule formation, and “reticulophagy”, a type of selective autophagy. Finally, we summarize the recent work that identified strategies by which ZIKV modulated these intrinsic antiviral responses.

## 1. Introduction

Zika virus (ZIKV) is a member of the *Flaviviridae,* which is comprised of enveloped viruses with a positive sense, single-stranded RNA genome [1]. Other members of this family include the mosquito-borne human pathogens dengue virus (DENV), yellow fever virus (YFV), Japanese encephalitis virus (JEV), and West Nile virus (WNV), as well as more distantly related blood-borne members such as the hepatitis C virus (HCV) [2]. While transmission by an *Aedes aegypti* mosquito vector is the primary route of ZIKV circulation, its ability to transmit through a vertical and sexual route makes this virus unique amongst the other mosquito-transmitted flaviviruses that cause diseases in humans [3,4,5].

Since its discovery in 1947, ZIKV infection in humans has been historically linked to sporadic cases of self-limiting symptoms such as fever, rash, and conjunctivitis [6]. After a 2013 outbreak in French Polynesia, ZIKV infection was associated with neurological complications in adults, including acute paralysis and the Guillain–Barré syndrome [7]. After the onset of a widespread 2015 epidemic in South America, ZIKV was identified as a causative agent of severe birth defects, such as microcephaly and cerebral calcifications, following in utero exposure to the virus [8,9,10]. While the increase in herd immunity over the past several years has reduced the number of human ZIKV infections worldwide, ZIKV remains a public health threat, given its potential for re-emergence [11].

ZIKV has a broad cell tropism in vitro, infecting human skin cells, such as dermal fibroblasts and epidermal keratinocytes; human myeloid cells, such as dendritic cells (DCs) and macrophages; and human progenitor cells of neuronal, placental, and testicular origin [12]. In order to gain access into specific target cells, including human microglia, astrocytes, and fetal endothelial cells, ZIKV is bound by Gas6, a ligand that recognizes phosphatidylserine on the viral membrane, which subsequently binds to its receptor Axl [13]. This bridging activity provided by Gas6 allows ZIKV to indirectly interact with Axl, facilitating entry into the cell [13,14]. In in vivo settings, ZIKV has been detected in the brain and spinal cord as well as in several cell types of both male and female reproductive tissue [12,15]. Upon entry into and uncoating within the host cell cytoplasm, the ~10.7 kb flavivirus RNA genome is translated into a single, large polyprotein that encodes three structural (C, prM/M, and E) and seven nonstructural (NS) proteins (NS1, NS2a, NS2b, NS3, NS4a, NS4b, and NS5) [16]. Polyprotein cleavage is mediated by host proteases, as well as the virally-encoded protease NS3. Protease activity of the NS3 protein requires its essential cofactor NS2b, and this proteolytically-active complex is often referred to as “NS2b3” [17]. The flavivirus NS proteins assemble at endoplasmic reticulum (ER)-membrane-derived vesicles to form the viral replication complex (RC), within which both NS3 and NS5 are central for the replication of the viral RNA genome, providing helicase and RNA-dependent RNA polymerase activity, respectively [18]. The flavivirus RNA genome is subjected to cleavage by cellular exoribonuclease XRN1 during viral replication. XRN1 stalling at distinct tertiary structures within the flavivirus genome results in the production of several incomplete degradation products termed subgenomic flavivirus RNAs (sfRNAs), which have been shown to promote viral replication and pathogenicity [19,20,21,22]. Following replication, the structural proteins C, prM/M, and E, in addition to several NS proteins, are involved in virion assembly at ER membranes and in viral egress from the cell [17]. Besides their central roles in the viral lifecycle, both the NS proteins and sfRNAs of ZIKV (and flaviviruses in general) modulate various innate and intrinsic pathways in the host.

Importantly, there is no approved vaccine or specific antiviral treatment available for ZIKV infection to date. Current drug design efforts primarily aim to target ZIKV proteins with enzymatic activities that are essential for virus replication, such as NS3 and NS5, while progress towards a ZIKV vaccine has been challenging due to the potential risk of neurological side effects and antibody-dependent enhancement of infection, a phenomenon that is best-characterized for DENV infection [23,24,25,26].

In this review, we summarize recently described roles of the mammalian antiviral type I interferon (IFN) system in restricting infection by ZIKV and related flaviviruses, as well as the molecular mechanisms by which these viruses evade the type I IFN response. Furthermore, we highlight recent work that showed that several host cell-intrinsic pathways play important roles in ZIKV restriction that, in turn, are modulated by ZIKV-encoded proteins. A detailed understanding of the mechanisms that ZIKV uses to evade or suppress host intrinsic or innate antiviral pathways might provide novel avenues for the development of antiviral drugs against ZIKV.

## 2. The Type I IFN System and Its Role in Restricting ZIKV Infection

**Figure 1:**
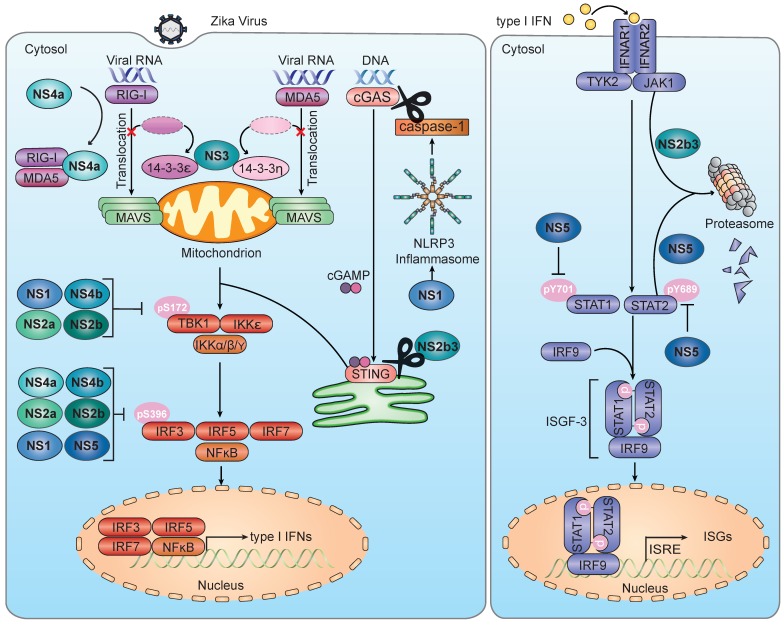
Zika-virus-mediated inhibition of the signaling pathways that lead to type I interferon (IFN) and IFN-stimulated genes (ISG) induction. During Zika virus (ZIKV) infection, a multitude of viral nonstructural (NS) proteins are involved in antagonism of signal transduction mediated by pattern-recognition-receptors (PRRs) leading to type I interferon (IFN) induction (left panel), as well as of IFN-mediated expression of IFN-stimulated genes (ISGs) (right panel). ZIKV NS3 interacts with the scaffold proteins 14-3-3ε and 14-3-3η and thereby prevents the translocation of the sensors RIG-I and MDA5 from the cytosol, where viral RNA binding occurs, to the mitochondria, where RIG-I and MDA5 transmit downstream signaling via the adaptor protein mitochondrial antiviral signaling (MAVS). Furthermore, ZIKV NS4a directly binds to both RIG-I and MDA5, thereby blocking their interaction with MAVS. Downstream of MAVS, ZIKV NS1, NS2a, NS2b, and NS4b have been found to reduce the phosphorylation of TBK1 at Ser-172, which suppresses TBK1 activation. ZIKV NS1, NS2a, NS2b, NS4a, NS4b, and NS5 have also been shown to inhibit IRF3 phosphorylation at Ser-396, which inhibits the downstream IFN transcription. Moreover, ZIKV NS1 has been shown to induce activation of the NLRP3 inflammasome, resulting in an enhanced cleavage of the DNA sensor cGAS by caspase-1. Downstream of the cGAS activation, ZIKV NS2b3 cleaves the adaptor protein STING, reducing its downstream signaling. In addition, several ZIKV proteins inhibit signaling downstream of the IFNα/β receptor (IFNAR). ZIKV NS2b3 degrades the kinase JAK1 in a proteasome-dependent manner, while ZIKV NS5 induces the proteasomal degradation of STAT2. Moreover, it has been shown that ZIKV NS5 inhibits the activating phosphorylation of STAT1 at Tyr-701 and of STAT2 at Tyr-689.

Early defense against viral pathogens is exerted by the innate immune system, which is comprised of pattern-recognition receptors (PRRs) that serve to detect conserved features of invading pathogens, universally called pathogen-associated molecular patterns (PAMPs) [27]. While many different PRRs exist in the mammalian species, they all serve to activate the transcription of type I IFNs (e.g., IFN-α subtypes and IFN-β) and other antiviral genes mediated by IFN-regulatory factors (IRFs) [27]. IFN-α/β secreted from the infected cell binds to the IFNα/β receptor (IFNAR) on either the same cell or neighboring uninfected cells, thereby mediating the autocrine and paracrine antiviral effects [28]. Activation of IFNAR induces a second transcriptional cascade that is mediated by signal transducer and activator of transcription factor 1 and 2 (STAT1 and STAT2) and results in the upregulation of many antiviral factors, generally known as IFN-stimulated genes (ISGs) [29] (Figure 1).

ZIKV replication has been shown to be inhibited by type I IFNs in several human cell types and in mouse models. For example, ZIKV replication was reduced in primary skin fibroblasts that were pre-treated with IFN-α or IFN-β, compared with cells that remained untreated [30]. Moreover, in primary human DCs, an increase in viral infectivity was observed when cells were treated with anti-IFNAR2 antibodies [31]. Mice lacking *IFNAR1* or *IRF 3*, *5*, and *7* developed more severe neurological disease compared to wild type (WT) mice [32]. Similarly, a mouse strain deficient in both subunits of *IFNAR* as well as *STAT2*^–/–^ mice were more susceptible to ZIKV infection and showed more severe ZIKV-associated pathology, compared to WT mice [33,34]. In addition, ZIKV infection of pregnant *IFNAR1*^–/–^ mice led to severe fetal growth restriction and fetal demise, compared to control mice [35,36]. Collectively, these observations highlighted the importance of the type I IFN response in restricting ZIKV infection both in human cells and in mice.

Restriction of ZIKV infection is mediated by a collection of ISGs, which are transcriptionally upregulated by IFNAR signaling in response to infection. ZIKV infection was shown to induce the expression of the ISGs *IFIT1-3*, *RSAD2/Viperin*, and *OAS1* in primary human DCs [31]. Furthermore, ZIKV infection of A549 (human lung carcinoma) cells resulted in enhanced expression of *IFN-β* and several ISGs (e.g., *MX1*, *IFIT1*, and *IFI44*), compared to mock infection [37]. Another study found that silencing or overexpression of the ISG protein IFITM3 enhanced or reduced ZIKV replication in HeLa (human cervical carcinoma) cells, respectively, while overexpression of another ISG protein, IFITM1, reduced ZIKV replication in A549 cells [38]. Together, these studies demonstrated that the IFN response and downstream ISG induction is activated following ZIKV infection as a means to control replication of the virus.

## 3. Innate Immune Sensing of Infection by ZIKV and Other Flaviviruses

*IFN-α/β* gene expression and subsequent upregulation of ISGs are triggered by an ensemble of PRRs following sensing of the invading pathogen. At least three major classes of PRRs contribute to the effective detection of flaviviruses—(1) toll-like receptors (in particular TLR 3 and 7), which sense viral RNA within the endosome; (2) retinoic acid-inducible gene I (RIG-I)-like receptors (RLRs) such as RIG-I itself and melanoma differentiation-associated protein 5 (MDA5), which recognize RNA species in the cytoplasm; and (3) cyclic GMP–AMP synthase (cGAS), which detects cellular dsDNA that is mislocalized during flavivirus infection [39,40,41,42]. Signaling induced by TLRs, RLRs, and cGAS converges on a common downstream signaling cascade that ultimately triggers transcription of IFNs and other cytokines, as well as ISGs. In brief, TLRs signal through a cytoplasmic Toll/interleukin-1 receptor (TIR) domain to recruit adaptor proteins MyD88, TRIF, or TRAM [43]. Upon activation by viral RNA, RIG-I and MDA5 translocate from the cytosol to mitochondria and mitochondria-associated membranes (MAMs) where they interact with mitochondrial antiviral signaling (MAVS) protein, causing it to form large protein “filaments”, which then signal further downstream [44]. cGAS, when activated, catalyzes the synthesis of cyclic GMP–AMP (cGAMP), which interacts downstream with stimulator of the IFN gene (STING) [45]. Signaling downstream of these adaptor proteins ultimately converges onto several kinases in the cytoplasm, such as TANK-binding kinase 1 (TBK1) and IκB kinase-ε (IKK-ε) or the IKK-α/β/γ complex, which activate the transcription factors IRF3/5/7 and NF-κB, respectively. Activation of these transcription factors through phosphorylation events allows them to upregulate the gene expression of many cytokines and chemokines, in addition to IFNs [46].

### 3.1. TLR Sensing of ZIKV and Other Flaviviruses

Endosomal TLRs, such as TLR3 and TLR7, are critical for innate detection of flavivirus infections, evidenced by work from many groups (reviewed in detail in [40]). For example, TLR3 has been shown to be required for induction of type I IFN during WNV infection of cortical neurons [47]. Furthermore, TLR7 and its downstream adaptor MyD88 are required for protection against lethal WNV infection in mice by controlling the viral burden in the brain [48,49]. In regards to DENV, overexpression of TLR3 was shown to inhibit DENV (serotype 2) replication in HEK293T cells, and co-localization of DENV RNA with TLR3 has been observed upon DENV (serotype 2) internalization [50]. Additionally, knockdown of TLR3 in Huh-7 (human hepatocellular carcinoma) cells or knockout of TLR3 in macrophages resulted in higher DENV (serotype 1) infection, compared to the control cells [51].

Similar to other flaviviruses, ZIKV RNA is believed to be sensed by endosomal TLR3 and TLR7, which are abundantly expressed on several cells relevant for ZIKV infection, including monocytes and DCs. Stimulation of monocyte-derived macrophages with a TLR7/8 agonist restricted ZIKV replication by inducing expression of several ISGs [52]. Additionally, silencing of TLR3 in human skin fibroblasts enhanced ZIKV RNA, although overall type I IFN mRNA expression was not affected [30]. In summary, these studies provided evidence for ZIKV restriction by TLR 3 and 7, although direct sensing of ZIKV PAMPs by these endosomal sensors has not yet been fully demonstrated.

### 3.2. RLR Sensing of ZIKV and Other Flaviviruses

Sensing of flaviviruses within the cell by the cytoplasmic sensors MDA5 and RIG-I has been well-characterized in the context of WNV and DENV infection. For example, WNV infection was enhanced in both *RIG-I*^–/–^ or *MDA5*^–/–^ mouse embryonic fibroblasts (MEFs), although at differing time points after infection [53]. Similarly, silencing of both RIG-I and MDA5 has been shown to enhance DENV (serotype 1) replication in Huh-7 cells [51]. Infection of *MAVS*^–/–^ mice revealed enhanced WNV replication and viral-induced lethality due to increased inflammation in the brain [54]. Similarly, *MAVS*^–/–^ mice were shown to be highly susceptible to DENV (serotype 2) infection and showed delayed IFN-α and IFN-β production [55].

The critical role of RLRs in ZIKV sensing and restriction has also been demonstrated by several studies. ZIKV-infected primary human skin fibroblasts transcriptionally upregulated *RIG-I* and *MDA5*, although silencing of these sensors did not significantly affect ZIKV replication in these cells [30]. Similarly, primary human DCs upregulated *RIG-I* and *MDA5* transcript and protein expression following ZIKV infection, and treatment with a RIG-I agonist derived from HCV RNA restricted ZIKV replication [31]. Furthermore, ZIKV replication was found to be markedly enhanced in *RIG-I*^–/–^, *MDA5*^–/–^*,* and *MAVS*^–/–^ human trophoblast cells—a cell type of the placental barrier—with the most enhanced replication observed in *MAVS*^–/–^ cells [56]. Accordingly, ZIKV-induced *IFN-β* gene expression was reduced in *RIG-I*^–/–^ and *MDA5*^–/–^ trophoblast cells, and completely abolished in *MAVS*^–/–^ cells [56], suggesting non-redundant roles of RIG-I and MDA5 in this cell type. Similarly, siRNA-mediated knockdown of either RIG-I or MDA5 in SVGA (human fetal astrocyte) cells reduced the expression of *IFN-β* and several ISGs following ZIKV infection and, accordingly, enhanced the viral titers. Furthermore, silencing of both RIG-I and MDA5 strongly enhanced ZIKV replication in this cell type [57]. Experiments in A549 cells showed that primarily RIG-I, but not MDA5, mediated the antiviral cytokine responses triggered by ZIKV infection [58]. Moreover, while transfection of total RNA isolated from ZIKV-infected A549 cells effectively activated an IFN-β-luciferase reporter in normal control cells or *MDA5*-deficient cells, reporter activation was strongly reduced in cells that lacked *RIG-I* or *MAVS* [37]. ZIKV RNA was also shown to directly associate with ectopically expressed RIG-I during infection, and these RIG-I-bound RNAs could induce the IFN-β reporter when re-transfected into uninfected cells [37]. These studies indicated that ZIKV RNA-PAMPs are sensed by both RIG-I and MDA5, whereby the relative contribution of the two sensors to IFN induction and ZIKV restriction differs according to the cell type.

### 3.3. cGAS-STING Sensing of ZIKV and Other Flaviviruses

In addition to RNA sensors, the cGAS-STING signaling axis has been shown to detect and restrict flaviviruses [41,42,59]. Mechanistically, it has been shown that mitochondrial DNA (mtDNA) that is leaked into the cytosol following DENV-induced perturbation of mitochondrial membranes leads to cGAS-STING activation and downstream IFN gene expression [41,42]. With regards to ZIKV infection, a recent study found enhanced ZIKV replication and reduced *IFN-β* induction in *cGAS^–/–^* THP1 (human monocytic) cells and peripheral blood mononuclear cells [60]. Furthermore, another study (described in detail below) demonstrated that ZIKV replication is enhanced in *STING^–/–^* human fibroblasts and that ZIKV actively antagonizes STING within the cGAS pathway [61].

In summary, recent studies have demonstrated that TLRs, RLRs, and the cGAS-STING pathway play an important role in innate immune sensing and control of the ZIKV infection. These sensors likely act together within the infected cell to detect a combination of ZIKV-derived PAMPs and host-derived molecules, or act in a cell type-specific manner. Thus, determining the relative contribution of these innate immune sensors to IFN/ISG induction and ZIKV restriction in different cell types and tissues will be important. Studies in knockout mice lacking specific sensors, especially TLRs, would provide important insight into the contribution of these sensors to ZIKV restriction and pathogenesis. Along these lines, the precise PAMPs recognized by PRRs during ZIKV infection are largely unknown. For example, it has not been fully established whether cGAS senses mtDNA during ZIKV infection, or perhaps other host DNA species. Moreover, the precise mechanism(s) whereby ZIKV induces cGAS-PAMPs during infection have not yet been elucidated, which will be an exciting avenue for future research.

## 4. Inhibition of IFN Induction by ZIKV and Other Flaviviruses

### 4.1. Mechanisms by Which DENV, WNV, and HCV Suppress IFN Production

Flaviviruses have evolved many mechanisms for antagonizing the host’s type I IFN response during infection [62]. These mechanisms include—(1) direct antagonism of the activation of specific PRRs; (2) sequestration of specific 14-3-3 scaffold proteins that control the subcellular localization of PRRs; and (3) inhibition of downstream signaling molecules in the IFN induction pathway (Figure 1).

A critical step in the activation of RIG-I is its Lys63-linked ubiquitination, mediated by the ubiquitin E3 ligase TRIM25 (and other E3 enzymes), which ultimately leads to RIG-I oligomerization and its binding to the adaptor protein MAVS [63,64]. The sfRNA of a specific epidemic strain of DENV (serotype 2, strain “PR-2”) has been shown to physically interact with TRIM25, preventing its activation and interaction with RIG-I [65]. Once RIG-I is activated by viral dsRNA and Lys63-polyubiquitin, it translocates from the cytosol to mitochondria and MAMs to induce MAVS-dependent downstream signaling [66]. The scaffold and chaperone protein 14-3-3ε, which belongs to the 14-3-3 protein family comprised of seven members in humans, facilitates the cytosol-to-mitochondrial translocation of RIG-I and its interaction with MAVS [67]. A conserved phosphomimetic motif, 64-Rx(E/D)P-67, within the NS3 protein of DENV and WNV binds to 14-3-3ε and thereby outcompetes RIG-I, ultimately blocking the translocation of RIG-I to mitochondria. Mutation of the conserved 14-3-3-binding motif of NS3 in the context of a recombinant DENV (serotype 2) resulted in enhanced cytokine induction and attenuated viral replication, compared with the parental virus [68]. Recent work has uncovered that the NS3 proteins of ZIKV strains (both from African and Asian lineages) encode a very similar 14-3-3-binding motif (64-RLDP-67) at the corresponding site as that of DENV and WNV NS3s, and thereby block RLR activation (as described in detail below), suggesting a conserved RLR-antagonistic mechanism between these three mosquito-transmitted flaviviruses [57]. In contrast to the described nonproteolytic RLR-inhibitory mechanism of DENV and WNV NS3, the HCV NS3 protein, together with its cofactor NS4a, directly cleaves MAVS, leading to its dislocation from mitochondria and inhibition of downstream signaling [69,70]. Furthermore, DENV NS4a has been reported to directly bind and sequester MAVS, blocking its interaction with RIG-I and downstream innate immune signaling [71], although the relative contribution of this evasion strategy to RIG-I inhibition remains to be determined. A recombinant WNV harboring mutations that prevented sfRNA formation was replication-deficient in WT cells and mice but replication-competent in cells and mice lacking both IRF3 and IRF7 or mice lacking IFNAR [72]. Moreover, transfection of JEV sfRNA into JEV-infected cells reduced the phosphorylation and nuclear translocation of IRF3, as well as reduced downstream IFN-β gene expression [73]. Although these studies strengthened the overall concept that sfRNAs of flaviviruses modulate innate immunity, future studies will be needed to fully define the molecular mechanisms of sfRNA-mediated immune evasion during flavivirus infection.

Given that the cGAS-STING axis also effectively restricts flavivirus infection [41,42,59], it is not surprising that these viruses have mechanisms to evade this pathway. DENV directly antagonizes both cGAS and STING through different mechanisms. The NS2b3 protease of DENV directly cleaves human—but not mouse—STING via its catalytic activity [74,75]. Furthermore, DENV (serotype 2 and 4) infection of primary human monocyte-derived dendritic cells (MDDCs) revealed a reduction in cGAS protein levels, compared to mock-infected controls [42]. Interestingly, expression of the DENV NS2b cofactor alone could inhibit cGAS/STING-dependent IFN-β promoter activity and reduce cGAS protein levels, despite its lack of intrinsic proteolytic activity [42]. During DENV infection, cGAS was targeted to membrane compartments that co-stained with autophagy markers, and treatment with autophagy and lysosome-acidification inhibitors restored the cGAS protein levels, demonstrating a role for autophagy in the degradation of cGAS by ZIKV NS2b [42]. In the case of HCV, its NS4b protein was shown to bind to STING, preventing its interaction with TBK1, ultimately dampening the IFN induction [76].

### 4.2. Mechanisms by Which ZIKV Inhibits IFN Production

Given the recent emergence of ZIKV as a human pathogen, innate immune evasion strategies mediated by this virus are not as well-understood as those utilized by other mosquito-borne flaviviruses. Whereas antagonism of the TLR sensing pathways by ZIKV remains largely elusive, recent studies uncovered some of the mechanisms by which ZIKV inhibits the RLR-MAVS and cGAS-STING pathways, or their shared downstream signaling proteins TBK1 and IRF3.

#### 4.2.1. Evasion of RLR-MAVS Signaling

Several studies have found that overexpression of specific ZIKV NS proteins inhibits IFN-β promoter activation stimulated by ectopic expression of RIG-I 2CARD, which is constitutively-active, and/or stimulated by RIG-I agonists in HEK293T cells [37,56,77,78,79]. ZIKV NS4a reduced IFN-stimulated response elements (ISRE)-promoter activation and IFN-β secretion triggered by transiently overexpressed RIG-I 2CARD or MDA5, but not by MAVS, TBK1, or a constitutively-active IRF3 mutant (IRF3/5D)—suggesting antagonism at the level of RLRs [56]. Ectopic expression of NS4a reduced the interaction of overexpressed MDA5 or RIG-I with endogenous MAVS in HEK293T cells [56], suggesting competitive binding as the mechanism of RLR inhibition (Figure 1). It remains to be defined how important the NS4a-mediated RLR-evasion mechanism is to the IFN antagonism within the context of authentic ZIKV infection.

Recent work showed that ZIKV is able to counteract RIG-I- and MDA5-mediated innate immunity by disrupting the interactions of both RLRs with their respective scaffold proteins, 14-3-3ε and 14-3-3η [57]. Previous studies established that these two 14-3-3 isoforms are important for RLR-mediated antiviral signaling. While the translocation of activated RIG-I from the cytoplasm to mitochondria is mediated by 14-3-3ε, the cytosol-to-mitochondrial relocalization of the sensor MDA5 is facilitated by 14-3-3η [67,80]. With regards to the ZIKV infection, knockdown of 14-3-3ε or 14-3-3η in SVGA cells strongly diminished the ZIKV-induced antiviral gene expression to similar levels as seen with RIG-I or MDA5 silencing. Conversely, ectopic expression of 14-3-3ε or 14-3-3η reduced ZIKV replication, suggesting that these two scaffold proteins restrict ZIKV infection by participating in the RLR-mediated IFN induction pathway. ZIKV NS3 was shown to bind to 14-3-3ε via a conserved motif, 64-RLDP-67, which mimics a classical, phosphorylated 14-3-3 binding motif of cellular proteins. The 64-RLDP-67 motif of ZIKV NS3 harbors a negatively-charged residue (D66) that mimics the central phospho-Ser/Thr residue found in the cellular motif. Binding of ZIKV NS3 to 14-3-3ε inhibits 14-3-3ε-mediated cytosol-to-mitochondria translocation of RIG-I [57], similar to the mechanism whereby DENV NS3 evades RIG-I signaling [68]. ZIKV NS3 was found to also bind 14-3-3η via its phosphomimetic motif and to inhibit the relocalization of MDA5 from the cytosol to mitochondria and subsequent antiviral signaling. A recombinant NS3 mutant ZIKV encoding a mutated 14-3-3-binding motif (64-RLDP-67 → 64-KIKP-67) showed enhanced cytokine and ISG induction and reduced viral replication, compared to the parental virus, supporting the importance of RLR/14-3-3 inhibition for IFN antagonism during the ZIKV infection [57].

#### 4.2.2. Evasion of the cGAS-STING Axis

With the knowledge of species-specific DENV-mediated antagonism of the cGAS-STING pathway [74], Ding et al. infected a panel of dermal fibroblasts isolated from humans, non-human primates, and mice with ZIKV and found that human and non-human primate fibroblasts were similarly permissive to ZIKV infection, while mouse fibroblasts were comparatively resistant [61]. Additionally, human fibroblasts exhibited reduced cGAMP-mediated IFN responses to ZIKV infection while mouse fibroblasts retained signaling [61]. Similar to the previously-described role of the DENV NS2b3 protease in STING antagonism [74], ZIKV NS2b3 cleaved both ectopically-expressed and endogenous human STING, but not mouse STING [61] (Figure 1). In addition to mouse STING, chimpanzee, rhesus macaque, and squirrel monkey STING were resistant to cleavage by ZIKV NS2b3, despite susceptibility of fibroblasts from these species to ZIKV infection [61]. Such species-specific susceptibility suggests that ZIKV might antagonize the cGAMP-STING axis in certain species via a mechanism other than NS2b3-mediated proteolytic cleavage of STING. Interestingly, a recent study provided evidence for cleavage of cGAS by caspase-1 downstream of ZIKV-mediated NLRP3 inflammasome activation in human THP1 cells [60]. Expression of ZIKV NS1 enhanced the NLRP3 inflammasome activation and stabilized caspase 1 by preventing its ubiquitination and degradation, suggesting a novel mechanism by which ZIKV evades the cGAS-STING sensing pathway [60].

#### 4.2.3. Evasion of TBK1 and IRF3

In addition to viral antagonism of PRRs or their adaptor proteins, ZIKV NS proteins were shown to inhibit innate immune signaling at the level of TBK1 and IRF3, as well [78,79,81]. Ectopic expression of NS1 and NS4b reduced phosphorylation of endogenous TBK1 at Ser-172 in Sendai virus-infected A549 cells, associated with a reduction in *IFN-β* expression [78]. NS1 and NS4b were found to bind ectopically-expressed TBK1, and a mechanism of inhibition of TBK1 oligomerization was proposed [78]. Likewise, ZIKV NS2a, NS2b, and NS4b proteins reduced RIG-I-2CARD-mediated TBK1 phosphorylation at Ser-172 in an overexpression context in the HEK293T cells [79]. However, whether antagonism of TBK1 phosphorylation or oligomerization by these ZIKV NS proteins occurred during more physiological conditions, in particular during native ZIKV infection, is yet to be fully confirmed.

Downstream of TBK1 phosphorylation and oligomerization, ZIKV NS5 was found to interact with both TBK1 and its activating binding partner TRAF6 in an overexpression context, resulting in a reduction of the TBK1–TRAF6 interaction. However, the functional consequence of this competitive binding on IFN induction was not investigated [81]. In a separate study that investigated the innate immune evasion of ZIKV NS proteins, ZIKV NS1 suppressed the RIG-I–mediated IFN-β promoter activation in a strain-specific manner [79]. Sequence analysis revealed an alanine to valine mutation at position 188 (A188V) in NS1 proteins that are capable of this IFN-β inhibition. Mechanistically, only NS1 proteins encoding valine-188 bound to TBK1 and suppressed RIG-I-2CARD-mediated phosphorylation of both TBK1 (at Ser-172) and IRF3 (at Ser-396). A recombinant ZIKV strain encoding the A188V substitution induced lower levels of IFN-β in A129 mice, showed enhanced neurovirulence in CD-1 neonate mice, and promoted a higher viremia in C57BL/6J mice than its WT counterpart, consequently demonstrating a physiological relevance for this mechanism of innate immune inhibition [79] (Figure 1).

In regards to IRF3 antagonism by ZIKV, it was found that the ectopic expression of full-length ZIKV NS5 reduced TBK1 and MAVS-mediated phosphorylation of ectopically-expressed IRF3 at Ser-396 in HEK293 cells [81]. Likewise, ZIKV NS2a, NS2b, NS4a, and NS4b, when overexpressed, reduced RIG-I 2CARD-mediated IRF3 phosphorylation at Ser-396 in HEK293T cells [79]. Furthermore, ZIKV NS5 was shown to bind endogenous IRF3 via its MTase domain and to inhibit IFN-β promoter activation stimulated by IRF3/5D [79].

In summary, several NS proteins of ZIKV have been reported to antagonize IFN production. However, many of these antagonism strategies are yet to be confirmed during native ZIKV infection, and their relevance for effective virus replication and immune evasion has to be tested in vivo. Studies investigating these IFN-antagonistic mechanisms in suitable mouse infection models might reveal cell-type- or tissue-specific roles for individual NS proteins, and provide important insight into ZIKV-mediated pathogenesis. Moreover, it will be important to evaluate the relative contribution of the proposed evasion mechanisms to IFN antagonism by ZIKV, potentially through generating mutant recombinant viruses in which specific ZIKV-host interactions are ablated.

While many studies have focused on NS protein-mediated IFN antagonism, our understanding of the role of the recently identified and characterized ZIKV sfRNAs [82] in modulating IFN induction is still rudimentary. For example, while transfection of ZIKV sfRNA reduced the IFN-β promoter activation mediated by agonists for RIG-I or MDA5 [83], the physiological roles and mechanisms of ZIKV sfRNAs in RLR inhibition during infection remain unknown.

Furthermore, although type I IFNs are the primary IFNs secreted by a wide variety of cells, several cell types, including trophoblasts of the placental barrier, have been shown to secrete type III IFNs (IFN-λ) to control the ZIKV infection [84]. Therefore, it has been proposed that ZIKV likely evades the action of these type III IFNs, in order to gain access to the fetal compartment, during vertical transmission. Investigating the coordinated mechanisms by which ZIKV evades both type I IFN and type III IFN responses might provide insight into ZIKV’s unique pathogenesis.

## 5. Inhibition of IFNAR Signaling by ZIKV and Other Flaviviruses

After production and secretion from the infected cell, IFN-α/β binds IFNAR1/2 to activate receptor-associated kinases Janus kinase (JAK) and tyrosine kinase 2 (TYK2) [46]. These kinases then phosphorylate signal transducer and activator of transcription factor 1 and 2 (STAT1 and 2), inducing their dimerization and subsequent assembly with IRF9 into the multimeric protein complex IFN-stimulated gene factor 3 (ISGF-3) [46]. ISGF-3 then translocates into the nucleus where it binds to IFN-stimulated response elements (ISRE) to drive the expression of a multitude of ISGs [29]. ISGs encode effector proteins that activate mechanisms to directly or indirectly counter viral infection. Some PRRs, their regulators (e.g., TRIM ubiquitin E3 ligases), and downstream signaling molecules are themselves ISGs, allowing for positive feedforward amplification within the infected cells, as well as sensitization of the neighboring uninfected cells [44]. Other ISGs encode for the antiviral restriction factors (e.g., APOBEC proteins, MxA, Viperin) that inhibit various steps in the virus lifecycle [29]. In turn, ZIKV and other flaviviruses are equipped with strategies to counteract the IFNAR signaling pathway (Figure 1).

### 5.1. Major Mechanisms by Which DENV, WNV and HCV Evade IFNAR Signaling

Besides antagonizing signaling that leads to IFN gene expression, flaviviruses have evolved many mechanisms to suppress signaling downstream of IFNAR1/2 activation. At the level of entry, WNV and likely also other flaviviruses induce the expression of suppressors of cytokine signaling (SOCS) 1 and 3 after binding to and activating TAM (Tyro3/Axl/Mer) receptors on dendritic cells; this ultimately results in SOCS1/3-mediated inhibition of JAK1 and further downstream signaling [85,86]. WNV infection has also been shown to reduce IFN-α-mediated phosphorylation of JAK1 and TYK2 and prevent nuclear accumulation of STAT1 and STAT2 downstream of the two kinases [87,88]. Many flaviviruses directly antagonize STAT1 and STAT2 signaling functions via the activity of their NS proteins. For example, DENV (serotype 2) NS4b has been shown to reduce ISRE-dependent gene expression and STAT1 phosphorylation, in response to IFN-β [89]. Similarly, HCV NS5a can bind STAT1 and inhibit its phosphorylation status in response to IFN-α, resulting in downregulated ISG expression [90]. The NS5 protein of the pathogenic NY99 strain of WNV has been shown to potently block STAT1 phosphorylation and translocation to the nucleus [88,91]. DENV NS5 was shown to bind to human STAT2, which reportedly blocks its phosphorylation and, thereby, its ability to transcriptionally upregulate ISGs [92]. Two elegant studies demonstrated that a proteolytically-processed form of DENV NS5 interacts with cellular UBR4 to degrade STAT2 in a proteasome-dependent manner, resulting in a reduced ISG induction [93,94]. Moreover, YFV NS5 was shown to interact with STAT2—dependent on the Lys63-linked ubiquitination of YFV NS5 (at Lys-6) by host E3 ligase TRIM23—allowing for the downstream inhibition of ISRE activation [95]. Given the previously-characterized and largely conserved flavivirus-encoded mechanisms to antagonize the JAK–STAT pathway, the IFNAR-signal inhibition has been the focus of many studies investigating immune evasion by ZIKV.

### 5.2. Evasion of the JAK–STAT Signaling Pathway by ZIKV

ZIKV infection has been shown to modulate innate immune signaling downstream of IFN production. For example, ZIKV infection of A549 cells has been associated with down-regulated expression of ISGs including *ISG15*, *IFIT1*, and *IFIT2* in response to IFN-β treatment, as compared to mock infection [78]. Likewise, ZIKV infection of Vero cells showed a striking reduction in *ISG15* and *OAS1* gene expression triggered by exogenous addition of IFN [96]. Furthermore, Huh-7 cells showed reduced expression of the ISGs *Viperin/RSAD2* and *IFIT1* during ZIKV infection, following poly(I:C) stimulation. Interestingly, cells of neural and placental origin also showed a reduced ISG response during ZIKV infection, compared to poly(I:C) stimulation, with the expression of *Viperin/RSAD2* being particularly diminished. Since overexpression of Viperin reduced ZIKV titers and, inversely, *Viperin/RSAD2*^–/–^ MEFs showed enhanced ZIKV permissiveness, the dampening effect of ZIKV on *Viperin/RSAD2* gene expression is likely a mechanism of immune evasion [97].

More specifically, ZIKV has been shown to antagonize IFNAR-induced signaling through a variety of mechanisms that include (1) JAK1 degradation, (2) inhibition of STAT1 and STAT2 phosphorylation, and (3) STAT2 degradation. The protein abundance of JAK1 was found to be reduced during ZIKV infection of the A549 cells, both in the absence and presence of IFN stimulation [78]. Furthermore, a reduction in JAK1 protein and ISG transcripts was observed in cells ectopically expressing the ZIKV NS2b3 protease, and treatment with proteasome inhibitor MG132 stabilized the JAK1 protein levels [78]. These data suggested that ZIKV NS2b3 induces JAK1 degradation by the proteasome, although additional studies are needed to corroborate these findings during more physiological conditions. Moreover, it will be important to determine the molecular mechanism of how JAK1 degradation is triggered by ZIKV NS2b3, as well as the relative contribution of this evasion strategy to successful ZIKV replication (Figure 1).

Downstream of JAK1, STAT1 and STAT2 must be phosphorylated in order to activate the expression of ISGs. In A549 cells and human DCs, ZIKV infection diminished the pools of endogenous phosphorylated STAT1 and STAT2 (p-STAT1/2), at residues Tyr-701 and Tyr-689, respectively, in the presence and absence of exogenous IFN treatment [31]. Likewise, endogenous STAT1 phosphorylation was reduced in ZIKV-infected A549 cells, and further, STAT1 nuclear translocation was slightly diminished in ZIKV-infected HeLa cells [78]. More specifically, Hertzog et al. showed ZIKV NS5-mediated reduction of endogenous p-STAT1 in HEK293T cells treated with exogenous IFN-β [37].

Similar to DENV, ZIKV has been shown to degrade human—but not mouse—STAT2 via its NS5 protein. Grant et al. found that ZIKV infection resulted in an MOI-dependent reduction in endogenous STAT2 protein abundance in Vero cells [96]. Ectopic expression of ZIKV NS5 induced degradation of endogenous STAT2 in several cell types, indicating that NS5 is sufficient to trigger STAT2 destabilization [96]. STAT2 degradation was shown to be mediated by the proteasome, as endogenous STAT2 protein levels were rescued by treatment with proteasome inhibitor MG132 [96]. In contrast to DENV NS5, ZIKV infection was able to induce endogenous STAT2 degradation even in *UBR4*^–/–^ cells, suggesting a unique mechanism of STAT2 inhibition by ZIKV NS5 [96]. Of note, the phenotype of ZIKV NS5-mediated STAT2 degradation was also described by two other independent studies [37,77] (Figure 1). Interestingly, recent work by Joyce et al. found that the E3 ubiquitin ligase PDLIM2 was up-regulated during HCV, DENV, and ZIKV infection in Huh7.5 cells [98]. *PDLIM2*^–/–^ Huh7.5 cells were more resistant to HCV, DENV, and ZIKV infection than parental cells [98]. This study also showed that knockout of *PDLIM2* resulted in less efficient IFN-α-mediated STAT2 degradation; however, STAT2 degradation during flavivirus infection was not investigated in *PDLIM2*^–/–^ cells [98].

In addition to NS protein-mediated IFNAR-signal inhibition, general evasion of IFNAR signal transduction has been observed during ZIKV entry, whereby the primary ZIKV receptor Axl dampened the innate immune response in CHME3 (human microglial) cells. When CHME3 cells were treated with an Axl antagonist, ZIKV infection resulted in enhanced *IFN-β* expression, suggesting that in addition to facilitating entry, Axl contributes to the suppression of the innate immune response to ZIKV infection [13]. Of note, the gene expression of the negative innate immune regulator *SOCS1* was also enhanced during treatment with an Axl antagonist [13]. On the other hand, it has been shown that treatment of Sertoli (human testes epithelium) cells with the same Axl antagonist slightly reduced the protein levels of SOCS1 and SOCS3, which correlated with an enhanced expression of some ISGs, such as *IFIT1* and *MxA* [99]. These results demonstrated a critical role for Axl in suppressing the innate immune response, similar to the TAM receptors during other flavivirus infections [85]. However, the mechanistic details of how Axl exerts inhibition of IFNAR signaling during ZIKV infection remain to be fully determined, which will be an exciting avenue for future research. Taken together, these studies support the general concept that several NS proteins of ZIKV work in concert to effectively inhibit IFNAR signaling in many different cell types. Future studies will be needed to determine the precise mechanisms by which ZIKV proteins antagonize the JAK–STAT pathway. For example, while several groups have shown reduced STAT1 phosphorylation during ZIKV infection, it is still unknown which ZIKV proteins or potential cellular factors are involved in this antagonistic approach. Furthermore, although STAT2 degradation has been shown to be mediated by ZIKV NS5, it is still unknown which domain(s) and potential motifs in the ZIKV NS5 are required for this function and further, how host factors, if any, are usurped by ZIKV NS5 to promote proteasome-mediated STAT2 degradation.

## 6. Manipulation of Other Antiviral Pathways by ZIKV

Several recent studies have reported on additional host pathways implicated in ZIKV restriction and, in turn, mechanisms whereby ZIKV is able to modulate these pathways. Below, we summarize the current data describing an important role for the cellular stress response, nonsense-mediated mRNA decay (NMD), and selective autophagy during ZIKV infection.

The cellular stress response induces the accumulation of dynamic cytoplasmic aggregates called stress granules (SGs), which are comprised of stalled mRNA-bound translation pre-initiation complexes and several protein factors [100]. During viral infection, stress granule formation can act anti-virally by sequestering translational machinery components, thereby preventing the effective translation of viral proteins [101]. Previous work has indicated that HCV manipulates stress granule components to promote replication [102], whereas WNV and DENV have been shown to antagonize SG formation [103]. However, only recent studies have described ZIKV-mediated inhibition of stress granule formation triggered by a variety of chemical ‘stressors’, including those that induce oxidative stress and ER stress [104,105,106]. Interestingly, recent data suggested that ZIKV might hijack and sequester SG components to promote viral replication [105], similar to HCV, although the precise mechanisms of how this benefits the virus are currently unknown.

Recent studies have provided evidence for ZIKV-mediated modulation of the NMD machinery. NMD is a cytoplasmic RNA surveillance pathway that functions to recognize and degrade aberrant RNAs (predominantly mRNAs) by recruiting Up-frameshift Protein 1 (UPF1) to nucleate assembly of the NMD machinery on target mRNAs [107]. Several RNA viruses, such as the alphaviruses Semliki Forest virus (SFV) and Sindbis virus (SINV), were found to be targeted by NMD, resulting in restricted viral replication [108]. On the other hand, the Human Immunodeficiency Virus-1 (HIV-1) has been shown to manipulate the NMD machinery in order to promote viral replication [109]. In general, cellular RNAs that act as NMD targets contain certain molecular features, such as (1) a long 3′UTR due to the presence of a pre-termination codon (PTC) or (2) the presence of an exon-junction complex (EJC) further than 50-nucleotides downstream of the termination codon (TC); however, it is largely unknown what viral RNA species are targeted by the NMD apparatus [110].

With regards to flaviviruses, HCV infection has been shown to cause accumulation of canonical NMD RNA targets, mediated by binding to and sequestering of the NMD component WIBG by the HCV core protein [111]. Similar to HCV, several canonical NMD RNA-targets were stabilized during ZIKV infection of Huh-7 cells and human neural progenitor cells (NPCs) [112]. Furthermore, ZIKV replication was enhanced upon silencing of UPF1 in NPCs, suggesting an antiviral role for NMD-mediated RNA degradation during ZIKV infection [112]. Ectopic expression of ZIKV C, which was found to interact with UPF1 by mass spectrometry analysis and co-immunoprecipitation, resulted in a reduction of nuclear UPF1 protein levels, although UPF1 is typically characterized as a cytoplasmic mediator of NMD [112]. Interestingly, in another elegant study, ZIKV C was also shown to interact with another NMD1 component, PYM1 [113]. Silencing of PYM1, UPF1, or an additional NMD protein, MAGOH, enhanced WNV, ZIKV, and DENV RNA levels. During WNV infection, the interaction of PYM1 with other members of the NMD machinery was diminished, although whether this also occurred during ZIKV infection remains to be tested [113].

Autophagy is a tightly-regulated cellular process wherein cytoplasmic materials are targeted for lysosomal degradation in a non-selective or selective manner [114]. Mechanistically, upstream signaling induces the repression of the autophagy regulator mammalian target of rapamycin complex 1 (mTORC1), resulting in the recruitment of autophagy factors that nucleate, elongate, and mature a double-membrane vesicle that engulfs cellular cargo [114]. This vesicle, termed the “autophagosome,” can subsequently fuse with the lysosome, resulting in acid hydrolysis and degradation of the engulfed components [114]. The process of autophagy has been characterized as both a proviral mechanism that can provide membranes for viral assembly, and as an antiviral mechanism that can directly target viral nucleic acids and proteins for degradation [115].

The role of autophagy during flavivirus infection is also multi-faceted, playing both proviral and antiviral roles during the viral lifecycle. For example, knockdown of autophagy machinery resulted in reduced HCV replication, correlated with enhanced IFN and ISG signaling in HCV-infected hepatocytes [116]. DENV (serotype 2) has been shown to activate autophagy to enhance RNA replication, facilitate virion maturation, and manipulate cellular metabolism in a proviral manner [117]. In contrast, chemical induction of autophagy has been shown to attenuate WNV genome replication and disease progression in WNV-infected mice [117].

Like other flaviviruses, ZIKV infection has been shown to induce autophagy in a variety of permissive cell types, including human skin fibroblasts [30], fetal neural stem cells (fNSCs) [118], human umbilical vein endothelial cells (HUVECs) [119], and human cytotrophoblasts [120]. Moreover, ZIKV replication was enhanced by treatment with autophagy-inducing chemical agents in human skin fibroblasts [30] and fNSCs [118]; inversely, ZIKV replication was reduced by treatment with pharmacological autophagy inhibitors in human skin fibroblasts [30], fNSCs [118], HUVECs [119], and human cytotrophoblasts [120]. Physiologically, ZIKV vertical transmission and placental and fetal damage were reduced in pregnant mice treated with autophagy inhibitors, as well as in pregnant mice that were hypomorphic for a key autophagy gene *Atg16L1,* suggesting that ZIKV might utilize autophagy to cross the placental barrier [120]. Collectively, these studies suggested that non-selective autophagy plays a pro-viral role during ZIKV infection.

While non-selective autophagy has been shown to be required for certain stages of the ZIKV lifecycle [30,118,119,120], selective autophagy at the ER, termed “reticulophagy”, has been implicated in restricting ZIKV replication [121]. Reticulophagy is a cellular process that regulates ER dynamics and protein quality control, and is induced by binding of autophagy machinery to the ER-localized reticulophagy receptors FAM134A, B, and C [122]. In a recent study, silencing of FAM134B resulted in enhanced replication of DENV (serotype 2) and ZIKV in human brain microvascular endothelial cells (HBMECs) [121]. Interestingly, a cleavage product of FAM134B was observed in cells expressing a DENV replicon. Ectopic expression of DENV or ZIKV NS2b3 induced cleavage of FAM134B, mediated by its proteolytic activity [121]. Additionally, overexpression of the NS2b3-mediated cleavage product of FAM134B reduced the number of FAM134B-localized autophagosomes at the ER, suggesting that reticulophagy might be induced as a host antiviral mechanism to restrict viral replication at the ER, while ZIKV in turn has evolved to cleave the reticulophagy receptor FAM134B via its NS2b3 protease to inhibit reticulophagy [121].

In summary, these studies provided evidence that stress granule formation, NMD, and reticulophagy have antiviral activity against ZIKV, and as such, ZIKV antagonizes these host processes to promote its replication. While the mechanistic details of viral targeting by these intrinsic pathways as well as of viral evasion, thereof, are currently not well-understood, the recent findings should stimulate more detailed investigation into these novel ZIKV-host interactions.

## 7. Conclusions and Future Perspectives

Since the emergence of ZIKV as an epidemic threat in 2015, impressive progress has been made in identifying and understanding ZIKV-mediated evasion of host antiviral pathways. While many viral mechanisms of innate immune antagonism have been elucidated, most studies have focused on those mediated by direct actions of ZIKV NS proteins. However, there is much to be discovered regarding potential host factors involved in these inhibitory mechanisms, as well as other means for evasion, such as ZIKV sfRNA-mediated antagonism of innate and intrinsic immunity. Interestingly, the majority of innate immune molecules antagonized by ZIKV have been previously shown to be targeted by related viruses, such as DENV and WNV. Along these lines, although the molecular mechanisms of ZIKV-mediated inhibition of the type I IFN response still need to be further elucidated, current data suggests that many of the evasion strategies are conserved among mosquito-transmitted flaviviruses. Since ZIKV infection in humans shows unique pathological features, it is expected that ZIKV has evolved unique innate evasion mechanisms, which will be an exciting avenue for future research. In particular, investigation of the combined role of type I IFN evasion and additional antiviral pathways that ZIKV is known to subvert, such as type III IFN production, might more clearly define the physiology of ZIKV infection at a molecular level. Furthermore, recent data have indicated that the activation of stress granule formation, NMD, and reticulophagy play an antiviral role during ZIKV infection. Additionally, elucidating the precise mechanisms by which these cellular pathways suppress ZIKV replication and contribute to the ZIKV-induced pathogenesis will be an exciting question in this emerging field. Importantly, whether ZIKV directly antagonizes these pathways as a means to subvert their antiviral activities, or whether ZIKV co-opts these cellular pathways to promote its replication is still not clearly understood. Moreover, it will be interesting to define whether these intrinsic pathways show cell-type- or viral strain-specific effects. Understanding the mechanisms of innate and intrinsic antiviral pathways in restricting ZIKV might identify potential molecular targets for therapeutic intervention of ZIKV infection. Elucidating novel host defense responses to ZIKV will stimulate research into the mechanisms of how ZIKV, in turn, evades or hijacks these pathways.

## References

[1] Song B.H., Yun S.I., Woolley M., Lee Y.M. (2017). Zika virus: History, epidemiology, transmission, and clinical presentation. J. Neuroimmunol..

[2] Mackenzie J.S., Gubler D.J., Petersen L.R. (2004). Emerging flaviviruses: The spread and resurgence of Japanese encephalitis, West Nile and dengue viruses. Nat. Med..

[3] Calvet G., Aguiar R.S., Melo A.S.O., Sampaio S.A., de Filippis I., Fabri A., Araujo E.S.M., de Sequeira P.C., de Mendonca M.C.L., de Oliveira L. (2016). Detection and sequencing of Zika virus from amniotic fluid of fetuses with microcephaly in Brazil: A case study. Lancet Infect. Dis..

[4] McDonald E.M., Duggal N.K., Brault A.C. (2017). Pathogenesis and sexual transmission of Spondweni and Zika viruses. PLoS Negl. Trop. Dis..

[5] Foy B.D., Kobylinski K.C., Chilson Foy J.L., Blitvich B.J., Travassos da Rosa A., Haddow A.D., Lanciotti R.S., Tesh R.B. (2011). Probable non-vector-borne transmission of Zika virus, Colorado, USA. Emerg. Infect. Dis..

[6] Weaver S.C., Costa F., Garcia-Blanco M.A., Ko A.I., Ribeiro G.S., Saade G., Shi P.Y., Vasilakis N. (2016). Zika virus: History, emergence, biology, and prospects for control. Antivir. Res..

[7] Cao-Lormeau V.M., Blake A., Mons S., Lastere S., Roche C., Vanhomwegen J., Dub T., Baudouin L., Teissier A., Larre P. (2016). Guillain-Barre Syndrome outbreak associated with Zika virus infection in French Polynesia: A case-control study. Lancet.

[8] Ventura C.V., Maia M., Dias N., Ventura L.O., Belfort R. (2016). Zika: Neurological and ocular findings in infant without microcephaly. Lancet.

[9] Driggers R.W., Ho C.Y., Korhonen E.M., Kuivanen S., Jaaskelainen A.J., Smura T., Rosenberg A., Hill D.A., DeBiasi R.L., Vezina G. (2016). Zika Virus Infection with Prolonged Maternal Viremia and Fetal Brain Abnormalities. N. Engl. J. Med..

[10] Mlakar J., Korva M., Tul N., Popovic M., Poljsak-Prijatelj M., Mraz J., Kolenc M., Resman Rus K., Vesnaver Vipotnik T., Fabjan Vodusek V. (2016). Zika Virus Associated with Microcephaly. N. Engl. J. Med..

[11] Pierson T.C., Diamond M.S. (2018). The emergence of Zika virus and its new clinical syndromes. Nature.

[12] Miner J.J., Diamond M.S. (2017). Zika Virus Pathogenesis and Tissue Tropism. Cell Host Microbe.

[13] Meertens L., Labeau A., Dejarnac O., Cipriani S., Sinigaglia L., Bonnet-Madin L., Le Charpentier T., Hafirassou M.L., Zamborlini A., Cao-Lormeau V.M. (2017). Axl Mediates ZIKA Virus Entry in Human Glial Cells and Modulates Innate Immune Responses. Cell Rep..

[14] Richard A.S., Shim B.S., Kwon Y.C., Zhang R., Otsuka Y., Schmitt K., Berri F., Diamond M.S., Choe H. (2017). AXL-dependent infection of human fetal endothelial cells distinguishes Zika virus from other pathogenic flaviviruses. Proc. Natl. Acad. Sci. USA.

[15] Olagnier D., Muscolini M., Coyne C.B., Diamond M.S., Hiscott J. (2016). Mechanisms of Zika Virus Infection and Neuropathogenesis. DNA Cell Biol..

[16] Mukhopadhyay S., Kuhn R.J., Rossmann M.G. (2005). A structural perspective of the flavivirus life cycle. Nat. Rev. Microbiol..

[17] Bollati M., Alvarez K., Assenberg R., Baronti C., Canard B., Cook S., Coutard B., Decroly E., de Lamballerie X., Gould E.A. (2010). Structure and functionality in flavivirus NS-proteins: Perspectives for drug design. Antivir. Res..

[18] Neufeldt C.J., Cortese M., Acosta E.G., Bartenschlager R. (2018). Rewiring cellular networks by members of the Flaviviridae family. Nat. Rev. Microbiol..

[19] Funk A., Truong K., Nagasaki T., Torres S., Floden N., Balmori Melian E., Edmonds J., Dong H., Shi P.Y., Khromykh A.A. (2010). RNA structures required for production of subgenomic flavivirus RNA. J. Virol..

[20] Pijlman G.P., Funk A., Kondratieva N., Leung J., Torres S., van der Aa L., Liu W.J., Palmenberg A.C., Shi P.Y., Hall R.A. (2008). A highly structured, nuclease-resistant, noncoding RNA produced by flaviviruses is required for pathogenicity. Cell Host Microbe.

[21] Goertz G.P., Abbo S.R., Fros J.J., Pijlman G.P. (2018). Functional RNA during Zika virus infection. Virus Res..

[22] MacFadden A., O’Donoghue Z., Silva P., Chapman E.G., Olsthoorn R.C., Sterken M.G., Pijlman G.P., Bredenbeek P.J., Kieft J.S. (2018). Mechanism and structural diversity of exoribonuclease-resistant RNA structures in flaviviral RNAs. Nat. Commun..

[23] Ekins S., Mietchen D., Coffee M., Stratton T.P., Freundlich J.S., Freitas-Junior L., Muratov E., Siqueira-Neto J., Williams A.J., Andrade C. (2016). Open drug discovery for the Zika virus. F1000Research.

[24] Tian H., Ji X., Yang X., Xie W., Yang K., Chen C., Wu C., Chi H., Mu Z., Wang Z. (2016). The crystal structure of Zika virus helicase: Basis for antiviral drug design. Protein Cell.

[25] Wang B., Thurmond S., Hai R., Song J. (2018). Structure and function of Zika virus NS5 protein: Perspectives for drug design. Cell. Mol. Life Sci..

[26] Wang Q., Yan J., Gao G.F. (2017). Monoclonal Antibodies against Zika Virus: Therapeutics and Their Implications for Vaccine Design. J. Virol..

[27] Takeuchi O., Akira S. (2010). Pattern recognition receptors and inflammation. Cell.

[28] Goubau D., Deddouche S., Reis e Sousa C. (2013). Cytosolic sensing of viruses. Immunity.

[29] Schneider W.M., Chevillotte M.D., Rice C.M. (2014). Interferon-stimulated genes: A complex web of host defenses. Annu. Rev. Immunol..

[30] Hamel R., Dejarnac O., Wichit S., Ekchariyawat P., Neyret A., Luplertlop N., Perera-Lecoin M., Surasombatpattana P., Talignani L., Thomas F. (2015). Biology of Zika Virus Infection in Human Skin Cells. J. Virol..

[31] Bowen J.R., Quicke K.M., Maddur M.S., O’Neal J.T., McDonald C.E., Fedorova N.B., Puri V., Shabman R.S., Pulendran B., Suthar M.S. (2017). Zika Virus Antagonizes Type I Interferon Responses during Infection of Human Dendritic Cells. PLoS Pathog..

[32] Lazear H.M., Govero J., Smith A.M., Platt D.J., Fernandez E., Miner J.J., Diamond M.S. (2016). A Mouse Model of Zika Virus Pathogenesis. Cell Host Microbe.

[33] Aliota M.T., Caine E.A., Walker E.C., Larkin K.E., Camacho E., Osorio J.E. (2016). Characterization of Lethal Zika Virus Infection in AG129 Mice. PLoS Negl. Trop. Dis..

[34] Tripathi S., Balasubramaniam V.R., Brown J.A., Mena I., Grant A., Bardina S.V., Maringer K., Schwarz M.C., Maestre A.M., Sourisseau M. (2017). A novel Zika virus mouse model reveals strain specific differences in virus pathogenesis and host inflammatory immune responses. PLoS Pathog..

[35] Yockey L.J., Jurado K.A., Arora N., Millet A., Rakib T., Milano K.M., Hastings A.K., Fikrig E., Kong Y., Horvath T.L. (2018). Type I interferons instigate fetal demise after Zika virus infection. Sci. Immunol..

[36] Yockey L.J., Varela L., Rakib T., Khoury-Hanold W., Fink S.L., Stutz B., Szigeti-Buck K., Van den Pol A., Lindenbach B.D., Horvath T.L. (2016). Vaginal Exposure to Zika Virus during Pregnancy Leads to Fetal Brain Infection. Cell.

[37] Hertzog J., Dias Junior A.G., Rigby R.E., Donald C.L., Mayer A., Sezgin E., Song C., Jin B., Hublitz P., Eggeling C. (2018). Infection with a Brazilian isolate of Zika virus generates RIG-I stimulatory RNA and the viral NS5 protein blocks type I IFN induction and signaling. Eur. J. Immunol..

[38] Savidis G., Perreira J.M., Portmann J.M., Meraner P., Guo Z., Green S., Brass A.L. (2016). The IFITMs Inhibit Zika Virus Replication. Cell Rep..

[39] Sabouri A.H., Marcondes M.C., Flynn C., Berger M., Xiao N., Fox H.S., Sarvetnick N.E. (2014). TLR signaling controls lethal encephalitis in WNV-infected brain. Brain Res..

[40] Suthar M.S., Aguirre S., Fernandez-Sesma A. (2013). Innate immune sensing of flaviviruses. PLoS Pathog..

[41] Sun B., Sundstrom K.B., Chew J.J., Bist P., Gan E.S., Tan H.C., Goh K.C., Chawla T., Tang C.K., Ooi E.E. (2017). Dengue virus activates cGAS through the release of mitochondrial DNA. Sci. Rep..

[42] Aguirre S., Luthra P., Sanchez-Aparicio M.T., Maestre A.M., Patel J., Lamothe F., Fredericks A.C., Tripathi S., Zhu T., Pintado-Silva J. (2017). Dengue virus NS2B protein targets cGAS for degradation and prevents mitochondrial DNA sensing during infection. Nat. Microbiol..

[43] Kawasaki T., Kawai T. (2014). Toll-like receptor signaling pathways. Front. Immunol..

[44] Chiang J.J., Davis M.E., Gack M.U. (2014). Regulation of RIG-I-like receptor signaling by host and viral proteins. Cytokine Growth Factor Rev..

[45] Cai X., Chiu Y.H., Chen Z.J. (2014). The cGAS-cGAMP-STING pathway of cytosolic DNA sensing and signaling. Mol. Cell.

[46] Ivashkiv L.B., Donlin L.T. (2014). Regulation of type I interferon responses. Nat. Rev. Immunol..

[47] Suthar M.S., Diamond M.S., Gale M. (2013). West Nile virus infection and immunity. Nat. Rev. Microbiol..

[48] Town T., Bai F., Wang T., Kaplan A.T., Qian F., Montgomery R.R., Anderson J.F., Flavell R.A., Fikrig E. (2009). Toll-like receptor 7 mitigates lethal West Nile encephalitis via interleukin 23-dependent immune cell infiltration and homing. Immunity.

[49] Szretter K.J., Daffis S., Patel J., Suthar M.S., Klein R.S., Gale M., Diamond M.S. (2010). The innate immune adaptor molecule MyD88 restricts West Nile virus replication and spread in neurons of the central nervous system. J. Virol..

[50] Tsai Y.T., Chang S.Y., Lee C.N., Kao C.L. (2009). Human TLR3 recognizes dengue virus and modulates viral replication in vitro. Cell. Microbiol..

[51] Nasirudeen A.M., Wong H.H., Thien P., Xu S., Lam K.P., Liu D.X. (2011). RIG-I, MDA5 and TLR3 synergistically play an important role in restriction of dengue virus infection. PLoS Negl. Trop. Dis..

[52] Vanwalscappel B., Tada T., Landau N.R. (2018). Toll-like receptor agonist R848 blocks Zika virus replication by inducing the antiviral protein viperin. Virology.

[53] Fredericksen B.L., Keller B.C., Fornek J., Katze M.G., Gale M. (2008). Establishment and maintenance of the innate antiviral response to West Nile Virus involves both RIG-I and MDA5 signaling through IPS-1. J. Virol..

[54] Suthar M.S., Ma D.Y., Thomas S., Lund J.M., Zhang N., Daffis S., Rudensky A.Y., Bevan M.J., Clark E.A., Kaja M.K. (2010). IPS-1 is essential for the control of West Nile virus infection and immunity. PLoS Pathog..

[55] Perry S.T., Prestwood T.R., Lada S.M., Benedict C.A., Shresta S. (2009). Cardif-mediated signaling controls the initial innate response to dengue virus in vivo. J. Virol..

[56] Ma J., Ketkar H., Geng T., Lo E., Wang L., Xi J., Sun Q., Zhu Z., Cui Y., Yang L. (2018). Zika Virus Non-structural Protein 4A Blocks the RLR-MAVS Signaling. Front. Microbiol..

[57] Riedl W., Acharya D., Lee J.H., Liu G., Serman T., Chiang C., Chan Y.K., Diamond M.S., Gack M.U. (2019). Zika Virus NS3 Mimics a Cellular 14-3-3-Binding Motif to Antagonize RIG-I- and MDA5-Mediated Innate Immunity. Cell Host Microbe.

[58] Esser-Nobis K., Aarreberg L.D., Roby J.A., Fairgrieve M.R., Green R., Gale M. (2019). Comparative Analysis of African and Asian Lineage-Derived Zika Virus Strains Reveals Differences in Activation of and Sensitivity to Antiviral Innate Immunity. J. Virol..

[59] Schoggins J.W., MacDuff D.A., Imanaka N., Gainey M.D., Shrestha B., Eitson J.L., Mar K.B., Richardson R.B., Ratushny A.V., Litvak V. (2014). Pan-viral specificity of IFN-induced genes reveals new roles for cGAS in innate immunity. Nature.

[60] Zheng Y., Liu Q., Wu Y., Ma L., Zhang Z., Liu T., Jin S., She Y., Li Y.P., Cui J. (2018). Zika virus elicits inflammation to evade antiviral response by cleaving cGAS via NS1-caspase-1 axis. EMBO J..

[61] Ding Q., Gaska J.M., Douam F., Wei L., Kim D., Balev M., Heller B., Ploss A. (2018). Species-specific disruption of STING-dependent antiviral cellular defenses by the Zika virus NS2B3 protease. Proc. Natl. Acad. Sci. USA.

[62] Gack M.U., Diamond M.S. (2016). Innate immune escape by Dengue and West Nile viruses. Curr. Opin. Virol..

[63] Gack M.U., Shin Y.C., Joo C.H., Urano T., Liang C., Sun L., Takeuchi O., Akira S., Chen Z., Inoue S. (2007). TRIM25 RING-finger E3 ubiquitin ligase is essential for RIG-I-mediated antiviral activity. Nature.

[64] Jiang X., Kinch L.N., Brautigam C.A., Chen X., Du F., Grishin N.V., Chen Z.J. (2012). Ubiquitin-induced oligomerization of the RNA sensors RIG-I and MDA5 activates antiviral innate immune response. Immunity.

[65] Manokaran G., Finol E., Wang C., Gunaratne J., Bahl J., Ong E.Z., Tan H.C., Sessions O.M., Ward A.M., Gubler D.J. (2015). Dengue subgenomic RNA binds TRIM25 to inhibit interferon expression for epidemiological fitness. Science.

[66] Kell A.M., Gale M. (2015). RIG-I in RNA virus recognition. Virology.

[67] Liu H.M., Loo Y.M., Horner S.M., Zornetzer G.A., Katze M.G., Gale M. (2012). The mitochondrial targeting chaperone 14-3-3epsilon regulates a RIG-I translocon that mediates membrane association and innate antiviral immunity. Cell Host Microbe.

[68] Chan Y.K., Gack M.U. (2016). A phosphomimetic-based mechanism of dengue virus to antagonize innate immunity. Nat. Immunol..

[69] Li X.D., Sun L., Seth R.B., Pineda G., Chen Z.J. (2005). Hepatitis C virus protease NS3/4A cleaves mitochondrial antiviral signaling protein off the mitochondria to evade innate immunity. Proc. Natl. Acad. Sci. USA.

[70] Meylan E., Curran J., Hofmann K., Moradpour D., Binder M., Bartenschlager R., Tschopp J. (2005). Cardif is an adaptor protein in the RIG-I antiviral pathway and is targeted by hepatitis C virus. Nature.

[71] He Z., Zhu X., Wen W., Yuan J., Hu Y., Chen J., An S., Dong X., Lin C., Yu J. (2016). Dengue Virus Subverts Host Innate Immunity by Targeting Adaptor Protein MAVS. J. Virol..

[72] Schuessler A., Funk A., Lazear H.M., Cooper D.A., Torres S., Daffis S., Jha B.K., Kumagai Y., Takeuchi O., Hertzog P. (2012). West Nile virus noncoding subgenomic RNA contributes to viral evasion of the type I interferon-mediated antiviral response. J. Virol..

[73] Chang R.Y., Hsu T.W., Chen Y.L., Liu S.F., Tsai Y.J., Lin Y.T., Chen Y.S., Fan Y.H. (2013). Japanese encephalitis virus non-coding RNA inhibits activation of interferon by blocking nuclear translocation of interferon regulatory factor 3. Vet. Microbiol..

[74] Aguirre S., Maestre A.M., Pagni S., Patel J.R., Savage T., Gutman D., Maringer K., Bernal-Rubio D., Shabman R.S., Simon V. (2012). DENV inhibits type I IFN production in infected cells by cleaving human STING. PLoS Pathog..

[75] Yu C.Y., Chang T.H., Liang J.J., Chiang R.L., Lee Y.L., Liao C.L., Lin Y.L. (2012). Dengue virus targets the adaptor protein MITA to subvert host innate immunity. PLoS Pathog..

[76] Ding Q., Cao X., Lu J., Huang B., Liu Y.J., Kato N., Shu H.B., Zhong J. (2013). Hepatitis C virus NS4B blocks the interaction of STING and TBK1 to evade host innate immunity. J. Hepatol..

[77] Kumar A., Hou S., Airo A.M., Limonta D., Mancinelli V., Branton W., Power C., Hobman T.C. (2016). Zika virus inhibits type-I interferon production and downstream signaling. EMBO Rep..

[78] Wu Y., Liu Q., Zhou J., Xie W., Chen C., Wang Z., Yang H., Cui J. (2017). Zika virus evades interferon-mediated antiviral response through the co-operation of multiple nonstructural proteins in vitro. Cell Discov..

[79] Xia H., Luo H., Shan C., Muruato A.E., Nunes B.T.D., Medeiros D.B.A., Zou J., Xie X., Giraldo M.I., Vasconcelos P.F.C. (2018). An evolutionary NS1 mutation enhances Zika virus evasion of host interferon induction. Nat. Commun..

[80] Lin J.P., Fan Y.K., Liu H.M. (2019). The 14-3-3eta chaperone protein promotes antiviral innate immunity via facilitating MDA5 oligomerization and intracellular redistribution. PLoS Pathog..

[81] Lin S., Yang S., He J., Guest J.D., Ma Z., Yang L., Pierce B.G., Tang Q., Zhang Y.J. (2019). Zika virus NS5 protein antagonizes type I interferon production via blocking TBK1 activation. Virology.

[82] Akiyama B.M., Laurence H.M., Massey A.R., Costantino D.A., Xie X., Yang Y., Shi P.Y., Nix J.C., Beckham J.D., Kieft J.S. (2016). Zika virus produces noncoding RNAs using a multi-pseudoknot structure that confounds a cellular exonuclease. Science.

[83] Donald C.L., Brennan B., Cumberworth S.L., Rezelj V.V., Clark J.J., Cordeiro M.T., Freitas de Oliveira Franca R., Pena L.J., Wilkie G.S., Da Silva Filipe A. (2016). Full Genome Sequence and sfRNA Interferon Antagonist Activity of Zika Virus from Recife, Brazil. PLoS Negl. Trop. Dis..

[84] Bayer A., Lennemann N.J., Ouyang Y., Bramley J.C., Morosky S., Marques E.T., Cherry S., Sadovsky Y., Coyne C.B. (2016). Type III Interferons Produced by Human Placental Trophoblasts Confer Protection against Zika Virus Infection. Cell Host Microbe.

[85] Bhattacharyya S., Zagorska A., Lew E.D., Shrestha B., Rothlin C.V., Naughton J., Diamond M.S., Lemke G., Young J.A. (2013). Enveloped viruses disable innate immune responses in dendritic cells by direct activation of TAM receptors. Cell Host Microbe.

[86] Rothlin C.V., Ghosh S., Zuniga E.I., Oldstone M.B., Lemke G. (2007). TAM receptors are pleiotropic inhibitors of the innate immune response. Cell.

[87] Guo J.T., Hayashi J., Seeger C. (2005). West Nile virus inhibits the signal transduction pathway of alpha interferon. J. Virol..

[88] Liu W.J., Wang X.J., Mokhonov V.V., Shi P.Y., Randall R., Khromykh A.A. (2005). Inhibition of interferon signaling by the New York 99 strain and Kunjin subtype of West Nile virus involves blockage of STAT1 and STAT2 activation by nonstructural proteins. J. Virol..

[89] Munoz-Jordan J.L., Sanchez-Burgos G.G., Laurent-Rolle M., Garcia-Sastre A. (2003). Inhibition of interferon signaling by dengue virus. Proc. Natl. Acad. Sci. USA.

[90] Kumthip K., Chusri P., Jilg N., Zhao L., Fusco D.N., Zhao H., Goto K., Cheng D., Schaefer E.A., Zhang L. (2012). Hepatitis C virus NS5A disrupts STAT1 phosphorylation and suppresses type I interferon signaling. J. Virol..

[91] Laurent-Rolle M., Boer E.F., Lubick K.J., Wolfinbarger J.B., Carmody A.B., Rockx B., Liu W., Ashour J., Shupert W.L., Holbrook M.R. (2010). The NS5 protein of the virulent West Nile virus NY99 strain is a potent antagonist of type I interferon-mediated JAK-STAT signaling. J. Virol..

[92] Mazzon M., Jones M., Davidson A., Chain B., Jacobs M. (2009). Dengue virus NS5 inhibits interferon-alpha signaling by blocking signal transducer and activator of transcription 2 phosphorylation. J. Infect. Dis..

[93] Ashour J., Laurent-Rolle M., Shi P.Y., Garcia-Sastre A. (2009). NS5 of dengue virus mediates STAT2 binding and degradation. J. Virol..

[94] Morrison J., Laurent-Rolle M., Maestre A.M., Rajsbaum R., Pisanelli G., Simon V., Mulder L.C., Fernandez-Sesma A., Garcia-Sastre A. (2013). Dengue virus co-opts UBR4 to degrade STAT2 and antagonize type I interferon signaling. PLoS Pathog..

[95] Laurent-Rolle M., Morrison J., Rajsbaum R., Macleod J.M.L., Pisanelli G., Pham A., Ayllon J., Miorin L., Martinez C., tenOever B.R. (2014). The interferon signaling antagonist function of yellow fever virus NS5 protein is activated by type I interferon. Cell Host Microbe.

[96] Grant A., Ponia S.S., Tripathi S., Balasubramaniam V., Miorin L., Sourisseau M., Schwarz M.C., Sanchez-Seco M.P., Evans M.J., Best S.M. (2016). Zika Virus Targets Human STAT2 to Inhibit Type I Interferon Signaling. Cell Host Microbe.

[97] Van der Hoek K.H., Eyre N.S., Shue B., Khantisitthiporn O., Glab-Ampi K., Carr J.M., Gartner M.J., Jolly L.A., Thomas P.Q., Adikusuma F. (2017). Viperin is an important host restriction factor in control of Zika virus infection. Sci. Rep..

[98] Joyce M.A., Berry-Wynne K.M., Dos Santos T., Addison W.R., McFarlane N., Hobman T., Tyrrell D.L. (2019). HCV and flaviviruses hijack cellular mechanisms for nuclear STAT2 degradation: Up-regulation of PDLIM2 suppresses the innate immune response. PLoS Pathog..

[99] Strange D.P., Jiyarom B., Pourhabibi Zarandi N., Xie X., Baker C., Sadri-Ardekani H., Shi P.Y., Verma S. (2019). Axl Promotes Zika Virus Entry and Modulates the Antiviral State of Human Sertoli Cells. MBio.

[100] Protter D.S.W., Parker R. (2016). Principles and Properties of Stress Granules. Trends Cell Biol..

[101] Montero H., Trujillo-Alonso V. (2011). Stress granules in the viral replication cycle. Viruses.

[102] Pager C.T., Schutz S., Abraham T.M., Luo G., Sarnow P. (2013). Modulation of hepatitis C virus RNA abundance and virus release by dispersion of processing bodies and enrichment of stress granules. Virology.

[103] Emara M.M., Brinton M.A. (2007). Interaction of TIA-1/TIAR with West Nile and dengue virus products in infected cells interferes with stress granule formation and processing body assembly. Proc. Natl. Acad. Sci. USA.

[104] Bonenfant G., Williams N., Netzband R., Schwarz M.C., Evans M.J., Pager C.T. (2019). Zika Virus Subverts Stress Granules To Promote and Restrict Viral Gene Expression. J. Virol..

[105] Hou S., Kumar A., Xu Z., Airo A.M., Stryapunina I., Wong C.P., Branton W., Tchesnokov E., Gotte M., Power C. (2017). Zika Virus Hijacks Stress Granule Proteins and Modulates the Host Stress Response. J. Virol..

[106] Amorim R., Temzi A., Griffin B.D., Mouland A.J. (2017). Zika virus inhibits eIF2alpha-dependent stress granule assembly. PLoS Negl. Trop. Dis..

[107] Hug N., Longman D., Caceres J.F. (2016). Mechanism and regulation of the nonsense-mediated decay pathway. Nucleic Acids Res..

[108] Balistreri G., Horvath P., Schweingruber C., Zund D., McInerney G., Merits A., Muhlemann O., Azzalin C., Helenius A. (2014). The host nonsense-mediated mRNA decay pathway restricts Mammalian RNA virus replication. Cell Host Microbe.

[109] Serquina A.K., Das S.R., Popova E., Ojelabi O.A., Roy C.K., Gottlinger H.G. (2013). UPF1 is crucial for the infectivity of human immunodeficiency virus type 1 progeny virions. J. Virol..

[110] Balistreri G., Bognanni C., Muhlemann O. (2017). Virus Escape and Manipulation of Cellular Nonsense-Mediated mRNA Decay. Viruses.

[111] Ramage H.R., Kumar G.R., Verschueren E., Johnson J.R., Von Dollen J., Johnson T., Newton B., Shah P., Horner J., Krogan N.J. (2015). A combined proteomics/genomics approach links hepatitis C virus infection with nonsense-mediated mRNA decay. Mol. Cell.

[112] Fontaine K.A., Leon K.E., Khalid M.M., Tomar S., Jimenez-Morales D., Dunlap M., Kaye J.A., Shah P.S., Finkbeiner S., Krogan N.J. (2018). The Cellular NMD Pathway Restricts Zika Virus Infection and Is Targeted by the Viral Capsid Protein. MBio.

[113] Li M., Johnson J.R., Truong B., Kim G., Weinbren N., Dittmar M., Shah P.S., Von Dollen J., Newton B.W., Jang G.M. (2019). Identification of antiviral roles for the exon-junction complex and nonsense-mediated decay in flaviviral infection. Nat. Microbiol..

[114] Glick D., Barth S., Macleod K.F. (2010). Autophagy: Cellular and molecular mechanisms. J. Pathol..

[115] Choi Y., Bowman J.W., Jung J.U. (2018). Autophagy during viral infection—A double-edged sword. Nat. Rev. Microbiol..

[116] Shrivastava S., Raychoudhuri A., Steele R., Ray R., Ray R.B. (2011). Knockdown of autophagy enhances the innate immune response in hepatitis C virus-infected hepatocytes. Hepatology.

[117] Ke P.Y. (2018). The Multifaceted Roles of Autophagy in Flavivirus-Host Interactions. Int. J. Mol. Sci..

[118] Liang Q., Luo Z., Zeng J., Chen W., Foo S.S., Lee S.A., Ge J., Wang S., Goldman S.A., Zlokovic B.V. (2016). Zika Virus NS4A and NS4B Proteins Deregulate Akt-mTOR Signaling in Human Fetal Neural Stem Cells to Inhibit Neurogenesis and Induce Autophagy. Cell Stem Cell.

[119] Peng H., Liu B., Yves T.D., He Y., Wang S., Tang H., Ren H., Zhao P., Qi Z., Qin Z. (2018). Zika Virus Induces Autophagy in Human Umbilical Vein Endothelial Cells. Viruses.

[120] Cao B., Parnell L.A., Diamond M.S., Mysorekar I.U. (2017). Inhibition of autophagy limits vertical transmission of Zika virus in pregnant mice. J. Exp. Med..

[121] Lennemann N.J., Coyne C.B. (2017). Dengue and Zika viruses subvert reticulophagy by NS2B3-mediated cleavage of FAM134B. Autophagy.

[122] Nakatogawa H., Mochida K. (2015). Reticulophagy and nucleophagy: New findings and unsolved issues. Autophagy.

